# The application of collagen in the repair of peripheral nerve defect

**DOI:** 10.3389/fbioe.2022.973301

**Published:** 2022-09-23

**Authors:** Xiaolan Li, Xiang Zhang, Ming Hao, Dongxu Wang, Ziping Jiang, Liqun Sun, Yongjian Gao, Ye Jin, Peng Lei, Yue Zhuo

**Affiliations:** ^1^ Department of Neurology and State Key Laboratory of Biotherapy, West China Hospital, Sichuan University, Chengdu, China; ^2^ School of Acupuncture-Moxi Bustion and Tuina, Changchun University of Chinese Medicine, Changchun, China; ^3^ Department of Oral and Maxillofacial Surgery, School and Hospital of Stomatology, Jilin University, Changchun, China; ^4^ Laboratory Animal Center, College of Animal Science, Jilin University, Changchun, China; ^5^ Department of Hand and Foot Surgery, The First Hospital of Jilin University, Changchun, China; ^6^ Department of Pediatrics, First Hospital of Jilin University, Changchun, China; ^7^ Department of Gastrointestinal Colorectal and Anal Surgery, China-Japan Union Hospital of Jilin University, Changchun, China; ^8^ Department of Pharmacy, Changchun University of Chinese Medicine, Changchun, China

**Keywords:** collagen, peripheral nerve injuries, peripheral nerve repair, nerve regeneration, nerve conduit

## Abstract

Collagen is a natural polymer expressed in the extracellular matrix of the peripheral nervous system. It has become increasingly crucial in peripheral nerve reconstruction as it was involved in regulating Schwann cell behaviors, maintaining peripheral nerve functions during peripheral nerve development, and being strongly upregulated after nerve injury to promote peripheral nerve regeneration. Moreover, its biological properties, such as low immunogenicity, excellent biocompatibility, and biodegradability make it a suitable biomaterial for peripheral nerve repair. Collagen provides a suitable microenvironment to support Schwann cells’ growth, proliferation, and migration, thereby improving the regeneration and functional recovery of peripheral nerves. This review aims to summarize the characteristics of collagen as a biomaterial, analyze its role in peripheral nerve regeneration, and provide a detailed overview of the recent advances concerning the optimization of collagen nerve conduits in terms of physical properties and structure, as well as the application of the combination with the bioactive component in peripheral nerve regeneration.

## 1 Introduction

Peripheral nerve injury (PNI) is a commonly encountered clinical issue with varying severity worldwide, reported in approximately 2.8% of all trauma patients (Noble et al., 1998). Despite relatively low incidence, the frequency of PNI is increasing worldwide and creating a severe economic burden (Grinsell and Keating, 2014). PNI is usually caused by direct mechanical trauma (Jiang et al., 2020a). Patients with peripheral neuropathy typically have severe motor or sensory deficits, innervation regions dysfunction, and neuropathic pain due to the destruction of the peripheral nerve plexus, nerve trunk, or its branches (Li et al., 2014; Sullivan et al., 2016).

Repairing severe PNI has always been one of the most challenging clinical practices in neurosurgery (Faroni et al., 2015). Large nerve defects are often difficult to recover due to the extremely slow process of axon regeneration (Holmquist et al., 1993). Currently, autologous nerve transplantation (ANT) remains the most efficacious microsurgical approach for repairing long peripheral nerve gaps (Ladak et al., 2011; Bassilios Habre et al., 2018; Choi et al., 2018). However, the application of ANT is plagued by damage to the donor site and limited donors (Nectow et al., 2012). Moreover, more than half of patients treated with ANT have failed to achieve successful recovery (Ruijs et al., 2005). Artificial nerve conduits, as an alternative to ANT, can act to connect the proximal and distal ends of the nerve defect, providing physical and biological guidance for axonal regeneration (Zhang et al., 2020). Various materials with excellent biocompatibility and biodegradability have been explored to prepare artificial nerve conduits, such as natural and synthetic biodegradable polymers (Boni et al., 2018).

Collagen is a natural polymer approved for clinical use as a nerve conduit (Boni et al., 2018). It is the main fibrous structural protein widely expressed throughout all organs and tissues and is therefore readily available (Karsdal et al., 2017). Further, collagen is known to exhibit low immunogenic properties and offers a porous structure, good biocompatibility, and biodegradability (Dong and Lv, 2016). Advanced understanding of these properties makes this natural polymer a novel biomaterial that can mimic the physiological property of nervous tissue and is widely used in peripheral nerve repair.

Previous preclinical and clinical studies have investigated the therapeutic effects of collagen-based nerve conduits on nerve regeneration (Okamoto et al., 2010; Cao et al., 2013; Lu et al., 2015). Functionalized collagen nerve conduits further enhanced nerve regeneration and functional recovery through improved physical properties, structural optimization, and incorporation of various bioactive components (Pereira Lopes et al., 2006; Yao et al., 2010a; Fujimaki et al., 2017). Collagen nerve conduits filled with growth factors-loaded collagen filaments have been successfully used to repair 35-mm nerve defects in large animal models (Cui et al., 2014; Yao et al., 2018), suggesting the potential applicability of collagen nerve conduits in bridging critical-sized defects in peripheral nerves. Given the breakthrough in peripheral nerve repair, this review summarizes the characteristics of collagen and its role in peripheral nerve regeneration, focusing on the research progress in using collagen as a nerve conduit biomaterial to repair peripheral nerve defects.

## 2 Characteristics of collagen as a biomaterial

### 2.1 The structure of collagen

**FIGURE 1:**
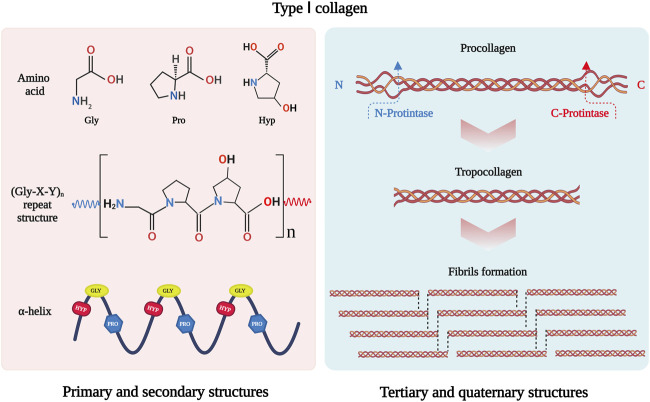
Schematic drawing of type I collagen structure. Gly-X-Y is the repeating peptide triplets. X and Y position is often occupied by proline and hydroxyproline. There α-chains are woven together to form procollagen with triple helix. Type I collagen is synthesized *in vivo* in the form of procollagen. The N- and C-terminal propeptides of procollagen are cleaved off by special enzymatic hydrolysis to form troprocollagen, and trigger spontaneous assembly to form fibrils. This figure was created with BioRender.com.

As the most abundant protein in mammals, collagen constitutes approximately 30% of total protein mass (Ricard-Blum, 2011). To date, 40 collagen genes have been identified to encode 29 collagen molecules, from collagen types I to XXIX (Sorushanova et al., 2019), which can be divided into fibril-forming collagens, fibril-associated collagens, network-forming collagens, anchoring fibrils, transmembrane collagens, basement membrane collagens and others with unique functions (Gelse et al., 2003). Among them, type I collagen is the major fibril-forming collagen in tissues and organs, and its wide range of sources makes it in great demand in tissue engineering. The collagen molecule consists of a triple-helical region and two non-helical regions at either end of the helix. The three left-handed α-chains are woven together around a central axis into a triple helix of procollagen. The amino acid sequence Gly-X-Y is the predominant repeating peptide triplets in trimeric collagen. As the minor amino acid, glycine residue is located in the center of the triple helix during the α chain assembly, while other bulky amino acid residues occupy the outer positions. The X and Y positions are usually occupied by proline and hydroxyproline, respectively (Gelse et al., 2003) (Figure 1). To stabilize the structure of the triple helix in collagen molecules, two hydrogen bonds per triplet are formed: one hydrogen bond is formed between the N-H group of Gly and the hydroxyl group of the adjacent chain X residue, another is an intramolecular hydrogen bond formed by hydroxyproline residues (Sorushanova et al., 2019). The non-collagenous domains flanking the central helical part are also essential collagen components in cross-linking and fibril formation (Koopmans et al., 2009). The triple helix structure of collagen prevents hydrolysis by most proteases and increases the stability of the collagen structure (Chung et al., 2004).

In addition, fibril-forming collagens can self-assemble into a fibril structure. The C- and N-propeptides of procollagen must be cleaved off by special enzymatic hydrolysis to initiate spontaneous assembly (Holmes et al., 2018). The fibril structure of collagen is achieved by covalent cross-linking, which depends on the hydroxylation state of telopeptide lysine residues (Yamauchi and Sricholpech, 2012). The fibrillar collagen has high mechanical resilience and plays a key role in providing mechanical support for connective tissues such as skin, tendons, and bones (Avila Rodriguez et al., 2018). It interacts with cells in connective tissues to regulate cell anchorage migration, proliferation, differentiation, and survival (Yang et al., 2004). As the major mechanical component in the extracellular matrix (ECM), its mechanical resilience structure gives collagen the ability to maintain the structural integrity of tissues and organs.

### 2.2 Biological characteristics of collagen

Collagen is considered a safe biomaterial due to its low immunogenicity. Despite concerns that it may induce an immune response as a material of animal origin, collagen is still considered a weak antigen (Furthmayr and Timpl, 1976). The presence of non-collagenous proteins (Delustro et al., 1986), cells and cell remnants (Esses and Halloran, 1983), and residues of cross-linking (Speer et al., 1980) all may be the cause of the immune response. Studies have shown that the terminal non-helical regions might be the sites for inducing immune responses (Michaeli et al., 1969; Lynn et al., 2004). To reduce the immune response of collagen, the terminal non-helical regions could be removed by proteolytic enzyme treatment, known as atelocollagen (Vizarova et al., 1994). The immune response can also be avoided by selecting an appropriate source of collagen and the method of extracting collagen.

The excellent biodegradability and biocompatibility are also advantages of collagen as a biomaterial. Although collagen has a tight and firm triple helical structure that prevents hydrolysis by most proteases (Fields, 2013), the collagen degradation process can be achieved by cleaving the intact collagen fibers through special proteolytic hydrolysis (Jablonska-Trypuc et al., 2016). The members of the matrix metalloprotease (MMP) family (Amar et al., 2017) and cathepsin K (Drake et al., 2017) were two well-known mammalian interstitial collagenolytic enzymes, which could recognize and bind collagen fibrils, then cleave the individual strands of the triple helix after unwinding the collagen fibril (Aguda et al., 2014; Sprangers and Everts, 2019). The generated collagen fragments could be taken up by micropinocytosis or receptor-mediated endocytosis and subsequently degraded by lysosomal cysteine proteases (Sprangers and Everts, 2019). Furthermore, collagen is non-cytotoxic and biocompatible with various cells, supporting cell growth and cell differentiation *in vitro* (Zhang et al., 2021a; Hinman et al., 2021). Based on these biological characteristics, collagens have potential properties for tissue engineering applications.

### 2.3 Sources and extraction of collagen

Collagen is abundant in sources due to its widely existed in the dermis, tendons and bones of animals (Dong and Lv, 2016). It can be extracted from human tissues such as peripheral nerve tissue (Fujii et al., 1986) or placenta (Spira et al., 1994), as well as from traditional animal sources, generally rat, bovine, porcine, and sheep (Vidal et al., 2020). Among them, rat-tail tendon collagen has been widely used in early work due to its high purity and relatively simple extraction process (Gonzalez-Masis et al., 2020). Recently, marine animals are an emerging source of collagen extraction, which has the advantages of low cost, easy availability, and low risk of disease transfer (Liu et al., 2022b).

As an insoluble macromolecular structure *in vivo*, animal-derived collagen could be extracted by various methods, usually using chemical reagents to extract collagen, such as dilute acetic acid, neutral salt solution and alkali treatment (Matinong et al., 2022). Dilute acetic acid is an ideal collagen extraction reagent that results in higher extraction and retains the triple helix of collagen with non-helical regions. It is often combined with enzymatic hydrolysis to cleave the highly cross-linked bonds (Sorushanova et al., 2019). In addition, physical methods such as ultrasonic and microwave irradiation (MWI) could improve collagen extraction by accelerating chemical reactions (Jin et al., 2019; Petcharat et al., 2021).

Although natural collagen has a wide range of sources and an evolving extraction process, animal-derived material still carries the risks of inducing immune responses, batch-to-batch variability and disease transmission (Lee et al., 2021). To avoid these concerns, a few safer methods have been proposed to synthesize collagen. Protein recombination is an emerging approach to the mass production of collagen. Various types of collagen could be produced in mammalian cells, insect cells, bacteria and yeast, transgenic animals, and transgenic plants (Dong and Lv, 2016; Avila Rodriguez et al., 2018). Advanced genetic engineering techniques facilitate efficient transgenic system for recombinant collagens co-expressed with both the alpha- and beta-subunits of a recombinant Prolyl 4-hydroxyprolin (P4H) to stabilize the triple-helix structure of collagen (Xu et al., 2011; Yu et al., 2014). In addition, collagen-like peptides can be achieved using synthetic strategies. This material resembles native collagen in its protein structure and folding (O'Leary et al., 2011; Kumar et al., 2014). Extensive sources of collagen and optimization of the extraction process ensure the great demand for collagen in tissue engineering.

## 3 The role of collagen in peripheral nerve regeneration

**FIGURE 2:**
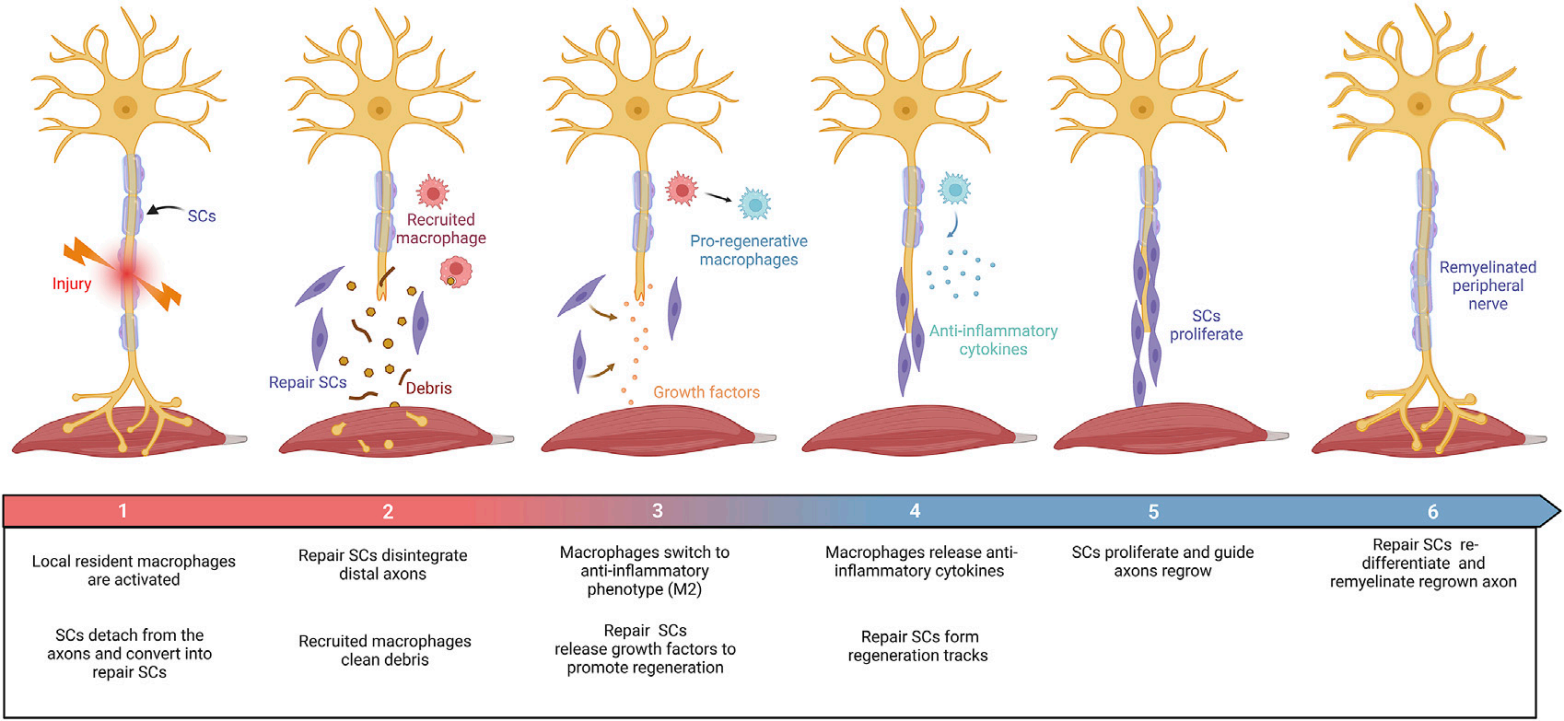
Schematic diagram summarizing the repair process in the PNI. Damage to peripheral nerves: (1) SCs were rapidly responded and converted into repair SCs. Local resident macrophages are activated. (2) Repair SCs disintegrate distal axons and recruit macrophages to clear debris. (3) Repair SCs release growth factors to promote axon regrowth. Macrophages switch to an anti-inflammatory phenotype (M2). (4) Repair SCs form regeneration tracks to guide axon regrowth. Macrophages secrete anti-inflammatory factors under the stimulation of the local injured microenvironment. (5) SCs proliferate and guide axon regeneration. (6) Finally, SCs transform into myelinating SCs and remyelinate the regenerated axon. This figure was created with BioRender.com.

Axons in the peripheral nervous system can regenerate after damage. Peripheral nerve repair is a diverse and complex process (Figure 2). Schwann cell (SC) behaviors (such as migration, proliferation, differentiation, and myelination), recruitment and polarization of macrophages, and release of growth factors are critical for the regeneration of peripheral nerves after injury (Bassilios Habre et al., 2018). Growing evidence suggests that several members of the collagen played key roles in the peripheral nervous system, where they affected the behaviors of SCs and maintained the physiological function of peripheral nerves (Sund et al., 2001; Chernousov et al., 2006; Rasi et al., 2010; Chen et al., 2014). For instance, collagen α4 type V has promoted SC adhesion, spreading, and migration by binding its N-terminal domain to heparin, mediated by syndecan-3, induced actin cytoskeleton assembly, tyrosine phosphorylation, and activation of Erk1/Erk2 protein kinases of SCs (Chernousov et al., 2001; Erdman et al., 2002). The absence of collagen VI in mice resulted in the hypermyelination of the peripheral nervous system, induced the activation of myelin-related signaling pathways, such as P-FAK, P-AKT, P-ERK1, P-ERK2, and P-p38, and accompanied by inhibition including P-JNK and P-c-Jun (Chen et al., 2014). These results consistently imply that collagens have functions in peripheral nerve regeneration.

**FIGURE 3:**
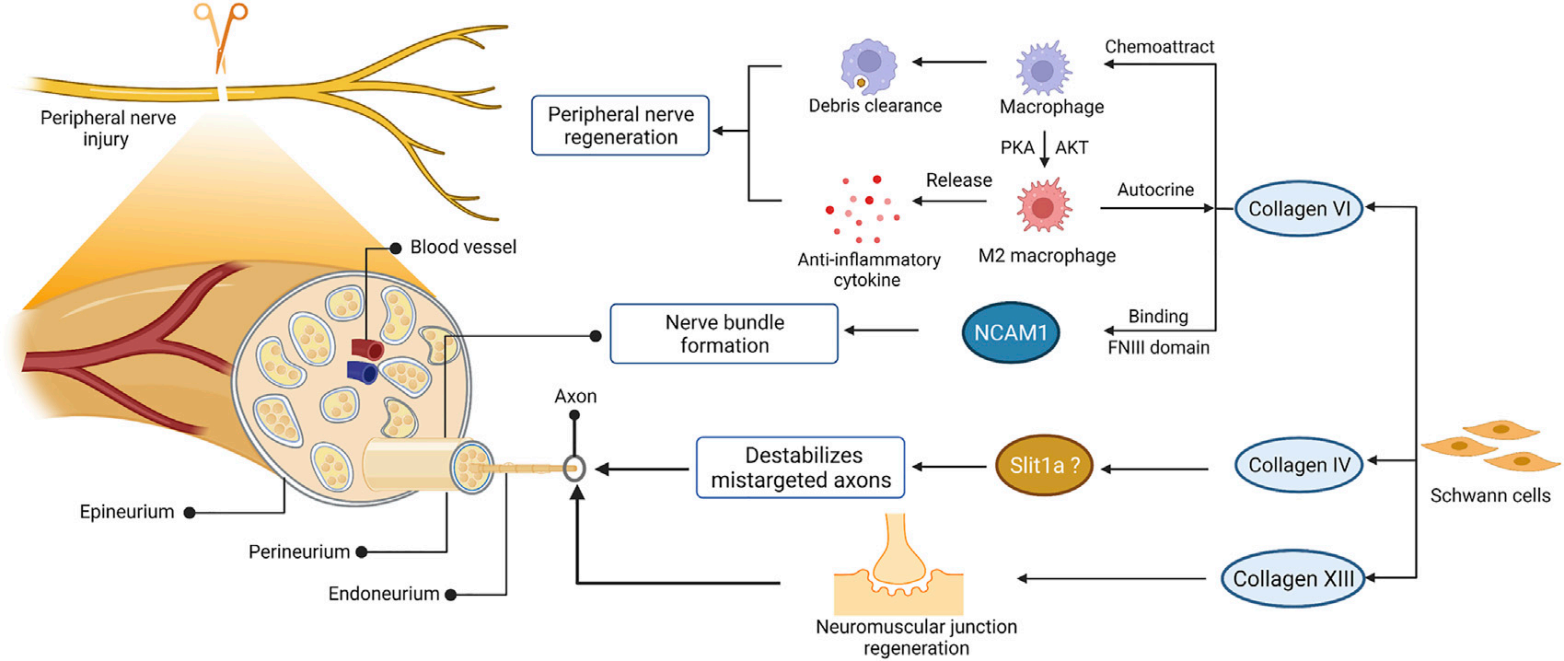
The role of collagen in peripheral nerve regeneration. The schematic representations of cross-sectional anatomy of the peripheral nerve and the role of collagen in peripheral nerve regeneration are shown on the left and right, respectively. This figure was created with BioRender.com.

Indeed, collagen contributes to the regeneration of peripheral nerves regeneration (Figure 3). After nerve injury, the expression of various collagen types was upregulated in peripheral nerves, such as collagen VI and IV (Gantus et al., 2006; Chen et al., 2015; Isaacman-Beck et al., 2015). Collagen VI was identified as a novel regulator for peripheral nerve regeneration that promoted macrophage migration and polarization via AKT and PKA pathways (Chen et al., 2015). The sustained release of collagen enhanced macrophage recruitment and polarized macrophages toward the M2 phenotype, thereby promoting nerve regeneration and functional recovery after sciatic nerve injury (Lv et al., 2017). Evidence has found that collagen VI also participated in regulating nerve bundle formation, mediated by direct binding to the FNIII domain of neural cell adhesion molecule 1 (NCAM1) in the extracellular space (Fang and Zou, 2021; Sun et al., 2022). As the substrate of lysyl hydroxylase 3, collagen4a5 destabilizes mistargeted axons to ensure target-selective regeneration *in vivo*, possibly via slit1a (Isaacman-Beck et al., 2015). Moreover, collagen XIII has been reported to affect synaptic integrity by binding the ColQ tail of acetylcholine esterase (Haronen et al., 2017). Further studies have confirmed its critical role in neuromuscular synapse regeneration and functional recovery after PNI (Zainul et al., 2018). Collectively, these observations suggest that collagen was required for the recruitment and polarization of macrophages, formation of nerve bundles, destabilization of mistargeted axons, and regeneration of neuromuscular synapses after PNI.

In particular, type I collagen is the most abundant and well-studied collagen among the various collagen types. It is the predominant type of collagen that exists in the peripheral nerve (Deal et al., 2012). After peripheral nerve injury, the endoneurial fibroblast is responsible for producing type I collagen (Siironen et al., 1992), which is thought to provide mechanical support for axonal growth and regeneration (Koopmans et al., 2009). In addition to the properties of natural polymers, such as excellent biocompatibility and degradability, type I collagen can be easily extracted from various animal sources and prepared into several physical forms according to special requirements. Thus, type I collagen is the most commonly used type of collagen in peripheral nerve reconstruction.

## 4 Design principles for advanced collagen-based nerve conduits

Based on the characteristics of collagen and its role in peripheral nerve development and regeneration, collagen is currently considered a suitable biomaterial for preparing nerve conduits to repair peripheral nerve injuries. However, their therapeutic effect is still not comparable to ANT. Thus, many attempts have been made to improve the physical properties, structure, and biological functions of collagen nerve conduits to mimic the *in vivo* microenvironment.

### 4.1 Improved physical properties

The physical properties of nerve conduits are known to strongly influence the regeneration process of peripheral nerves after transplantation (Salvatore et al., 2014). The nerve conduit must be able to provide mechanical support for the regenerated axon under pressure from surrounding tissues, and its degradation rate should also match the regeneration rate of the peripheral nerve (Harley et al., 2004). However, despite attracting great interest in nerve tissue engineering due to its excellent biological properties, natural collagen is limited by its poor mechanical strength and faster degradation rate *in vivo* (Itoh et al., 2002). Damage to assembly structure and natural cross-linking in the extraction process will lead to poorer mechanical properties and stability of extracted collagen than collagen in its natural state (Gu et al., 2019a). Therefore, cross-linked strategies were developed to improve the mechanical strength and stability of collagen (Bozkurt et al., 2009). Increased intermolecular cross-links between collagen molecules reduce the degrees of freedom of its α-chains, thereby improving the thermal stability of collagen (Harrington and Von Hippel, 1961). Besides, the cleavage site of collagen can be masked by intermolecular cross-linking and enhance the ability of collagen to resist enzymatic degradation (Aldahlawi et al., 2016).

Based on the characteristics of various cross-linking methods, which can be defined into three classes: chemical, physical and enzymatic cross-linking. Chemical cross-linking is the most effective and widely used strategy due to its uniform and high degree of cross-linking. However, residues of chemical agents in collagen molecule, such as the cross-linking agent glutaraldehyde (GA), which was widely used in previous studies, might lead to cytotoxicity (Salvatore et al., 2014). The later proposed 1-ethyl-3-(3-dimethylaminopropyl) carbodiimide/N-hydroxysuccinimide (EDC/NHS) became the most widely used cross-linking method for collagen nerve conduits due to the removal of activated intermediates (Yao et al., 2010a; Yao et al., 2010b; Cao et al., 2013). Physical cross-linking is generally considered a simple and safe method, such as UV irradiation, MWI, and dehydro-thermal treatment (DHT) (Itoh et al., 2002; Ahmed et al., 2004; Haugh et al., 2009). It can avoid the introduction of exogenous toxic chemicals into tissues and cytotoxicity. Moreover, enzymatic cross-linking is promising as an effective method for collagen due to its precise kinetics of reaction and non-cytotoxic. Compared with chemical and physical cross-linking, enzymatic cross-linking is the most expensive strategy. In general, different cross-linking methods have different effects on the degree of cross-linking and safety performance (Itoh et al., 2002; Ahmed et al., 2005; Salvatore et al., 2014), and the time and temperature during the cross-linking process will also affect the physical properties of collagen (Harley et al., 2004). *In vivo* studies using nerve guides with varying degrees of cross-linking showed that the degree of cross-linking could significantly affect the ability of peripheral nerve regeneration (Bozkurt et al., 2012). Thus, for peripheral nerve injuries with varying degrees, mechanical properties and degradation rates need to be accurately controlled by cross-linking methods.

### 4.2 Various physical forms of collagen in nerve conduits

**FIGURE 4:**
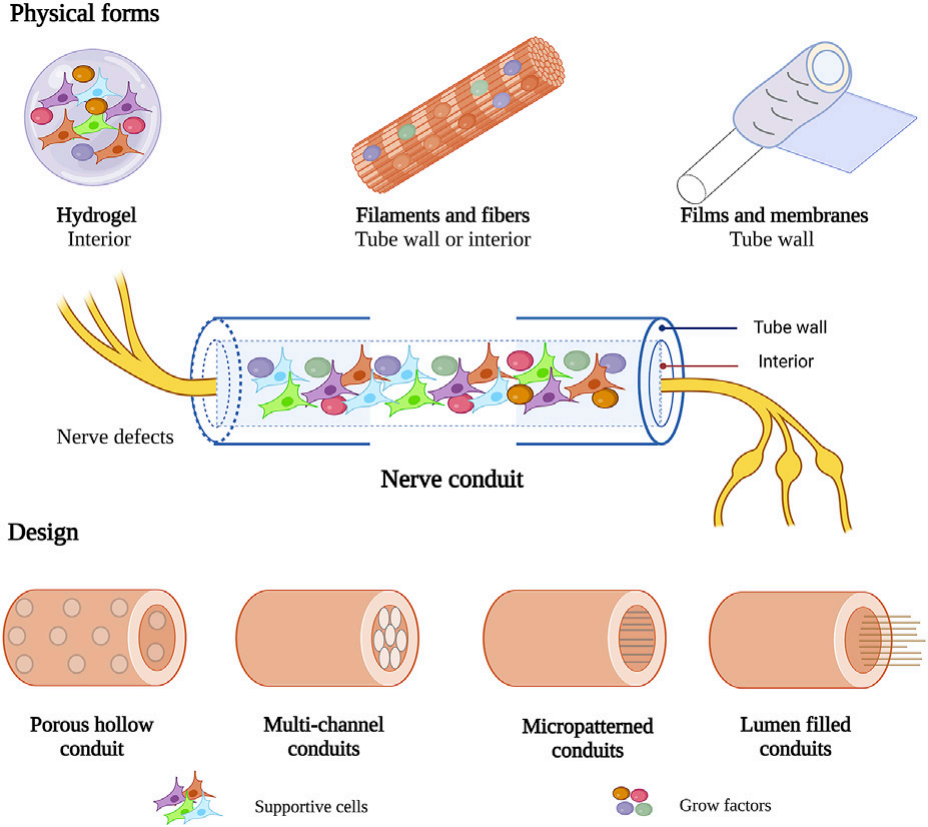
Various physical forms and design of collagen in nerve conduit. This figure was created with BioRender.com.

To optimize the structure of nerve conduits, collagen in various physical forms was used to prepare the walls or as an internal filler of nerve conduits for peripheral nerve repair, including hydrogels, filaments and fibers, films and membranes (Figure 4). Different physical forms of collagen have their advantages. To achieve better therapeutic effect, functional composite nerve conduits are often used in studies to combine the advantages of different physical forms of collagen.

#### 4.2.1 Hydrogels

Hydrogels are semi-solid structures containing networks formed by water-insoluble polymers, which serve as a three-dimensional (3D) substrate for nerve cell culture and have shown promise as scaffolds for nerve tissue engineering. As the ubiquitous structural component in the ECM, type Ⅰ collagen can self-assemble into a fibrillar gel under physiological temperature and pH, providing cell adhesion, support, and structural networks (Drzewiecki et al., 2014). Its fibrillar structure and major role in ECM make collagen a suitable hydrogel material for mimicking the properties of neural tissue *in vivo*. Compared with conventional cultures, collagen hydrogels are more physiologically relevant to the structure and ECM *in vivo*, could provide adequate space for neural cell growth and migration, and ensure efficient cell-cell and cell-ECM communication (Antoine et al., 2014). The physical properties of collagen hydrogel, such as elasticity and mechanical strength, can also be tuned to match the particular physiological property of nerve cells or tissue (Mori et al., 2013; Antoine et al., 2014). Changes in the mechanical properties of collagen hydrogels significantly affected the morphological patterns, phenotypic progression and behaviors of nerve cells (East et al., 2009; Ribeiro et al., 2012; Balasubramanian et al., 2016). Therefore, these excellent biological and physiological properties of collagen hydrogels make them suitable as an inner filler for nerve conduits to provide a suitable growth environment for loaded cells. Stem cell-based therapy has received much attention in cell-transplantation therapy for PNI. The soft 3D-collagen hydrogel drove the conversion of mesenchymal stem cells (MSCs) into neuronal lineage by the activation of NOTCH and PI3K-Akt signaling pathway (Zhang et al., 2021a; He et al., 2021), as well as contributed to the enhanced expression of neural phenotypes and release of neurotrophic factors in umbilical cord blood (UCB) cells and MSCs (Lee et al., 2014; Park et al., 2014). Further *in vivo* studies using collagen hydrogels loaded with human MSCs (Zhang et al., 2021a), SCs (Muangsanit et al., 2020), and bioactive molecules (Midha et al., 2003; Masand et al., 2012; Gonzalez-Perez et al., 2017; Samadian et al., 2019) significantly improved functional recovery and axonal regeneration. These results suggest that collagen hydrogel promoted the regeneration of peripheral nerves by providing support for the supportive cells and bioactive molecules within nerve conduits.

#### 4.2.2 Filaments and fibers

Collagen fibers have often been used as nerve conduits’ inner filler to load growth factors (Hayakawa et al., 2021). A previous study reported a new method for preparing linear-ordered collagen (LOC) fibers and considered it a good nerve guidance material (Lin et al., 2006). Compared to extracted collagen, collagen fibers prepared from aponeuroses could maintain the natural fiber structure and avoid the introduction of exogenous toxic compounds (Lin et al., 2006). Subsequent studies have achieved the combination of linear-ordered collagen scaffold (LOCS) and neurotrophins by fusing a collagen-binding domain (CBD) (Han et al., 2009) or laminin-binding domain (LBD) (Cao et al., 2011) in the N-terminal of neurotrophins. The sustained delivery of neurotrophins may provide a more favorable microenvironment for axon regeneration. These functional composite nerve conduits exhibited favorable mechanical properties and strongly promoted nerve regeneration and functional recovery in rodent and large animal models (Cao et al., 2013; Cui et al., 2014; Ma et al., 2014a; Yao et al., 2018). As well as an inner architecture for nerve guides, collagen fibers can be used to produce fibrous nerve conduits using many novel techniques. Single collagen fibers exhibited poor mechanical properties, while collagen microfibers fabricated by a microfluidic approach had excellent mechanical stability and thermal characteristics and showed excellent biocompatibility and growth-directing properties on the culture of neuronal NG108-15 cells, which indicates potential applications in peripheral nerve repair (Haynl et al., 2016). Moreover, collagen could be blended with synthetic polymers to improve mechanical properties and produce nanofibrous using electrospinning, 3D nanofibrous nerve conduits with highly longitudinal aligned nanofibers provided an optimal environment for axonal regeneration (Ouyang et al., 2013). Collagen fibers with additional topographical guidance further facilitated the neuronal response to injury and showed superior guidance for cell growth and neurite extension along the fibers (Hoffman-Kim et al., 2010).

#### 4.2.3 Films and membranes

To avoid the destruction of the physical structure of natural collagen during the extraction process, several methods have been reported to prepare collagen membranes with oriented fibers structure from tendons (Dai et al., 2012; Alberti and Xu, 2013). The prepared collagen membranes could be wound around a tubular rod to form a nerve conduit, retaining the native triple-helix structure and strength of collagen fibers (Alberti et al., 2014). This collagen conduit supported directional nerve growth, resulting in a more mature SC phenotype, and may promote the formation of the correct connections in re-growing axons (Alberti et al., 2014). Collagen conduits prepared from flow-oriented collagen fibrils exhibited highly porous and mechanically robust properties. *In vivo* results confirmed the directing role of its micro-patterned structure in the adherence and proliferation of sprouting axons (Ahmed et al., 2004). Furthermore, a bilayer collagen membrane has been used to fabricate nerve conduits (Zhuang et al., 2016). This collagen membrane was composed of loosely arranged collagen fibers in the inner layer and dense tight fibers in the outer layer. Its unique structure could effectively prevent the growth of soft tissue into the conduits without affecting cell adhesion and the exchange of nutrients and metabolites, which is similar to the design of nerve conduits with gradient pores (Cerri et al., 2014). These collagen membranes with an oriented-fiber structure contributed to the directed growth of axon regeneration.

### 4.3 Design of collagen nerve conduits

Collagen nerve conduits have been used in nerve regeneration for over 40 years (Colin and Donoff, 1984). It has evolved from single conduits to functional composite nerve conduits. Several designs of collagen-based nerve conduits have been proposed to promote axon regeneration in PNI, including hollow conduits, multichannel conduits, micropatterned conduits, and lumen-filled conduits (Figure 4).

#### 4.3.1 Hollow conduits

The hollow collagen conduit is the simplest design in the manufacturing process of nerve conduits. Its limitations include poor permeability of nutrient and growth factors and an inability to guide axonal growth (Yao et al., 2010b; Vijayavenkataraman, 2020). To optimize the structure of the hollow conduit, several modifications have been reported to make hollow collagen nerve conduits with porous properties, such as unidirectional freezing (Bozkurt et al., 2009), electrospinning (Ouyang et al., 2013), and freeze-drying (Lowe et al., 2016). The collagen scaffold with longitudinal guidance channels guided neurite outgrowth from adult DRG (Bozkurt et al., 2007) and facilitated the formation of ‘‘bands of Büngner’’ *in vitro* (Bozkurt et al., 2009). *In vivo* studies using this collagen scaffold seeded with SC could effectively repair a 20-mm-long sciatic nerve gap in rats using an epineurial sheath tube (EST) technique (Bozkurt et al., 2011), with somatosensory and motor neurons extending their axons across the implant, similar to autografts (Bozkurt et al., 2012; Bozkurt et al., 2016). Moreover, 3D nanofibrous collagen nerve conduits formed by electrospinning not only have sufficient mechanical properties to support cell growth but also guide SC growth and axonal alignment during regeneration (Ouyang et al., 2013).

#### 4.3.2 Multichannel conduits

Multichannel conduits with structural stability were designed to limit axonal dispersion and provide better guidance for nerve growth. A novel multistep process was reported to generate multichannel collagen conduits using cylindrical molds, followed by cross-linking with EDC and NHS (Yao et al., 2010a; Yao et al., 2010b). *In vitro* studies showed that 4- and 7-channel nerve conduits possessed favorable properties for nerve regeneration applications (Yao et al., 2010a). Further evaluation of nerve morphometry and the accuracy of regeneration in a 1-cm sciatic nerve gap indicated that 4-channel collagen conduits were considered the most suitable structure for peripheral nerve regeneration (Yao et al., 2010b). Furthermore, collagen conduits with a multichannel structure facilitated the loading of neurotrophic factors and supportive cells (Yao et al., 2013; Liu et al., 2021). The limitation of axonal dispersion on the multichannel structure combined with supportive cells or growth factors will be more beneficial to the regeneration of peripheral nerves.

#### 4.3.3 Micropatterned conduits

Neurons could respond to topography in specific ways (Hoffman-Kim et al., 2010). Many studies have proved that porous scaffolds with micropatterns were important for arranging cells in predesigned locations and directing the regeneration of complex networks (Rieu et al., 2019; Yu et al., 2021; Zhang et al., 2022). Several novel approaches have been reported to prepare micropatterned collagen for PNI. A spinning technique could be used to produce highly porous tubular constructs. The micropatterned collagen scaffold (MPCS) has been reported to be prepared by a spinning technique (Harley et al., 2006), characterized by a radially oriented pore structure in the wall of the conduit. The special structure with a small outer and large inner pore ensured that cells could grow and migrate inside the conduits without infiltrating outside. *In vivo* studies using MPCS confirmed the enhanced nerve morphogenesis in a 10-mm sciatic nerve traumatic injury, and gene expression profiles revealed that known genes related to PNS regeneration were regulated with MPCS (Cerri et al., 2014). Furthermore, the gradient collagen micropatterns played different regulatory effects on SCs (Li et al., 2019), which indicate that micropatterns of collagen can be extended to different patterned structures to promote nerve regeneration.

#### 4.3.4 Lumen-filled conduits

Collagen nerve conduits filled with fibers or hydrogel were more suitable for cell growth and incorporation of bioactive molecules (Li et al., 2018). LOC fibers were most widely filled in collagen nerve conduits, facilitating cell migration and guided nerve growth along the fibers. The collagen conduits filled with longitudinal collagen filaments successfully repaired a 30-mm sciatic nerve gap in dogs (Okamoto et al., 2010). The combination of this functional collagen-based nerve conduit with neurotrophin was further investigated (Cao et al., 2011; Shi et al., 2014): filled LOC fibers contributed to the sustained release of neurotrophin and strongly enhanced nerve regeneration and functional recovery (Cao et al., 2013; Ma et al., 2014a; Lu et al., 2015). Hydrogel was beneficial to the sustained release of neurotrophin. The axonal regeneration and functional recovery were similar to the autologous group using GelMA hydrogel-loaded glial cell-line derived neurotrophic factor (GDNF) (Zhuang et al., 2016). Hydrogels could also provide an excellent environment for the growth of supportive cells. Collagen conduits filled with fibrin-agarose hydrogels for loading adipose-derived mesenchymal stem cells (ADMSCs) resulted in enhanced functional recovery and nerve regeneration in the rat sciatic nerve gap (Carriel et al., 2013). The design of the inner filling of collagen nerve conduits provides a more permissive environment for cell growth and the binding of neurotrophic factors, thereby promoting axonal regeneration.

### 4.4 Collagen-based nerve conduits combined with bioactive components

#### 4.4.1 Supportive cells

Collagen-based nerve conduit loaded with supportive cells is an effective way to promote nerve regeneration and functional recovery. Research mainly focuses on the beneficial effects of SCs and stem cells on PNI (Table 1).

**TABLE 1:** Supportive cells fill in collagen material to repair PNI.

Cell type	Mechanism	Strategies	Outcomes	Nerve	Animal models
Schwan cells (SCs)	• Recruit macrophages (Zigmond and Echevarria 2019)	• Collagen conduit with inner collagen skeleton (Keilhoff et al. 2003)	• Axon regeneration similar to autograft (Bozkurt et al. 2012, Burks et al. 2021)	• Sciatic nerve (Keilhoff et al. 2003, Bozkurt et al. 2012, Berrocal et al. 2013, Burks et al. 2021)	• Rat (Keilhoff et al. 2003, Bozkurt et al. 2012, Berrocal et al. 2013, Burks et al. 2021)
	• Secrete neurotrophic factors (Madduri and Gander, 2010)	• Collagen-based nerve guide with longitudinal guidance channels (Bozkurt et al. 2009, Bozkurt et al. 2012)	• Improved myelination (Berrocal et al. 2013, Burks et al. 2021)		
	• Form Büngner to guide the axonal regrowth (Nocera and Jacob, 2020)	• NeuraGen 3D collagen matrix conduits (Burks et al. 2021)	• Decreased muscle atrophy (Burks et al., 2021)		
	• Re-myelinate the regenerated axon (Nocera and Jacob, 2020)				
Bone marrow-derived mesenchymal stem cells (BDMSCs)	• Differentiate into SC-like cells (Mimura et al. 2004, Cai et al. 2017)	• Biodegradable collagen tube (Pereira Lopes et al. 2006)	• Improved myelination and motor function recovery (Pereira Lopes et al. 2006)	• Sciatic nerve (Pereira Lopes et al. 2006, Ladak et al. 2011)	• Mice (Pereira Lopes et al. 2006)
	• Increase production of trophic factors (Novikova et al., 2011)	• Collagen conduits filled with differentiated mesenchymal stem cells (MSCs) (Ladak et al. 2011)	• Improved neurite outgrowth *in vitro* and motoneurons regeneration *in vivo* (Ladak et al. 2011)		• Rat (Ladak et al. 2011)
Adipose-derived mesenchymal stem cells (ADMSCs)	• Differentiate into SC-like cells (Liu et al., 2022a)	• Collagen nerve guide conduits containing a natural fibrin-agarose material (Carriel et al., 2013)	• Improved myelination and recovery of sensory and motor functions (Carriel et al., 2013)	• Sciatic nerve (Carriel et al., 2013)	• Rat (Carriel et al., 2013)
	• Secrete neurotrophin (Lopatinaet al., 2011)				
Human umbilical cord mesenchymal stem cells (hUC-MSCs)	• Differentiate into SC-like cells (Xiao and Wang, 2015)	• Collagen conduit filled with longitudinally aligned collagenous fibers (Cui et al., 2018)	• Improved regeneration and functional recovery (Cui et al., 2018)	• Sciatic nerve (Cui et al., 2018)	• Dog (Cui et al., 2018)
	• Secrete various neurotrophic factors and deposit extracellular matrix proteins (Guo et al. 2015)				
Gingiva-derived mesenchymal stem cells (GMSCs)	• Differentiate into neural crest stem-like cells (NCSC) (Zhang et al. 2018), neural progenitor-Like Cells ((iNPCs) (Zhang et al. 2017) and Schwann cell precursor-like (SCP) cells (Zhang et al. 2021a)	• 3D-collagen hydrogel (Zhang et al. 2021a)	• Improved functional recovery and axonal regeneration (Zhang et al. 2021a)	• Facial nerve (Zhang et al. 2021a)	• Rat (Zhang et al. 2021a)
	• Upregulation of NOTCH3 signaling pathway (Zhang et al. 2021a)				
Dental pulp stem cells (DPSCs)	• Differentiate into NCSC and SC-like cells (Al-Zer and Kalbouneh, 2015)	• Collagen conduits (Yamamoto et al. 2016)	• Improved myelination and revascularization (Yamamoto et al. 2016)	• Sciatic nerve (Yamamoto et al. 2016)	• Rat (Yamamoto et al. 2016)
Olfactory ensheathing cells (OECs)	• Remove degenerating axons via phagocytosis (Wewetzer et al. 2005, Bock et al. 2007)	• Collagen-chitosan conduits filled with a “PFTBA-OECs” enriched fibrin hydrogel (Zhu et al., 2014)	• Overcome the hypoxic status within nerve scaffolds (Zhu et al., 2014)	• Sciatic nerve (Zhu et al. 2014; Roche et al. 2017)	• Rat (Zhu et al. 2014, Roche et al. 2017, Gu et al. 2019b)
	• Secrete neurotrophic factors (Brouch et al., 2001)	• Biphasic collagen and laminin functionalized hyaluronic acid-based nerve guidance conduit (Roche et al., 2017)	• Improved axonal regeneration and functional recovery (Zhu et al., 2014, Gu et al., 2019b)	• Facial nerve (Gu et al., 2019b)	
		• Collagen sponge (Gu et al., 2019b)	• Improved clinical and electrophysiological outcomes (Roche et al., 2017)		
Neural stem cells (NSCs)	• Differentiate into cells of the neural lineage and SC-like cells (Tong et al., 2010)	• NT-3-supplemetned HA-Collagen composite conduit (Zhang et al., 2008)	• Re-innervations of damaged facial nerve (Zhang et al., 2008)	• Facial nerve (Zhang et al., 2008, Ma et al. 2017)	• Rabbit (Zhang et al., 2008)
	• Secrete various neurotrophic factors (Llado et al., 2004)	• Rat-tail collagen gel with the anchored bFGF (Ma et al., 2017)	• Functional recovery and nerve growth similar to autograft (Ma et al., 2017)		• Rat (Ma et al., 2017)

##### 4.4.1.1 Schwann cells

SCs are the principal glial cells that support neurons in the peripheral nervous system (PNS) and play a central role in peripheral nerve repair. After nerve transection, SCs from both proximal and distal nerve stumps dedifferentiated into repair SCs through the reprogramming process, followed by the release of neurotrophins (Madduri and Gander, 2010; Nocera and Jacob, 2020), proliferated and migrated into the nerve bridge, formed bands of Büngner to guide axon regeneration, and finally re-differentiated into myelinating SCs and remyelinated the regenerated axons (Jessen et al., 2015; Nocera and Jacob, 2020). Thus, additional SC-seeding may provide a unique opportunity to promote axonal regeneration further.

The development of techniques for culturing SCs from adult rat and human materials make it possible to add SCs to neural grafts (Keilhoff et al., 1999; Keilhoff et al., 2000; Calderon-Martinez et al., 2002). Collagen nerve guide conduits containing SCs were used for a longer nerve gap. *In vivo* results showed that collagen nerve conduits supported SCs behaviors, such as adhered, survived, and proliferated on the inner surface, which suggests that collagen conduits seeded with SCs may be applied to repair extended nerve gaps (Keilhoff et al., 2003). In addition, the SCs’ behaviors were regulated by the micropattern of collagen (Li et al., 2019). Collagen scaffolds with longitudinally oriented channels not only supported SC growth and migration but also guided SCs to align longitudinally along the channels, their framework highly supportive of peripheral nerve regeneration (Bozkurt et al., 2009; Bozkurt et al., 2012; Zhang et al., 2013). Further optimization of collagen nerve guide structure could provide a more suitable environment for SC growth and migration, thereby promoting axonal regeneration.

##### 4.4.1.2 Stem cells

The limitations of *in vitro* culture of SCs led to the investigation of other cell types that may present similar SC phenotypes or provide neurotrophic support to promote axonal regeneration. Stem cells represent a class of cells capable of self-renewal, proliferation, and differentiation (Thomson et al., 1998). Previous studies have shown that stem cells promoted nerve regeneration through differentiating into specific cell types (Kingham et al., 2007; Arthur et al., 2008; Berrocal et al., 2013) and sustained releasing of neurotrophic factors (Sowa et al., 2012). These properties make stem cell-based therapies widely used in preclinical peripheral nerve repair. The roles of various types of stem cells in peripheral nerve regeneration have been investigated in collagen-based nerve conduits (Ladak et al., 2011; Sowa et al., 2012; Ma et al., 2017).

The most comprehensively studied stem cells in collagen nerve conduits are MSCs (Zhang et al., 2021b), which could differentiate into SC-like cells (Dezawa et al., 2001; Zhang et al., 2021a). MSCs exhibited an equivalent efficacy on neurite outgrowth and axon regeneration as SCs (Ladak et al., 2011). Various derived MSCs have been filled in collagen nerve conduits to investigate the efficacy in nerve regeneration, such as bone marrow-derived mesenchymal stem cells (BMSCs) (Pereira Lopes et al., 2006), ADMSCs (Carriel et al., 2013), human umbilical cord mesenchymal stem cells (hUC-MSCs) (Cui et al., 2018), and dental pulp stem cells (Yamamoto et al., 2016). Further *in vivo* studies using stem cells-loaded collagen conduits significantly improved the nerve regeneration and recovery of sensory and motor functions (Carriel et al., 2013), and the longitudinally aligned collagenous fibers loaded with hUC-MSCs resulted in greater myelin sheath formation and functional recovery (Cui et al., 2018).

Olfactory ensheathing cells (OECs) are specialized glial cells between SCs and astrocytes. The therapeutic efficacy of various collagen-based nerve conduits seeded with OEC for peripheral nerve injury has been investigated (Li et al., 2010; Guerout et al., 2011). Nerve regeneration and functional recovery were significantly improved using OEC therapy (Gu et al., 2019b). Increased nerve fibers, myelinated fibers, and myelin area were observed in the OEC group (Goulart et al., 2016). Increased oxygen levels within the nerve conduit contributed to the enhanced therapeutic effect of OECs on nerve regeneration (Zhu et al., 2014).

Furthermore, neural stem cells (NSCs) can differentiate into neurons and promote locomotor recovery in spinal cord-injured mice (Cummings et al., 2005). Various collagen scaffolds loaded with NSCs have shown remarkable therapeutic effects in spinal cord injury (Cummings et al., 2005; Jiang et al., 2020b). To improve the therapeutic effect after transplantation, basic fibroblast growth factor (bFGF) was anchored on heparinized collagen nerve conduits to promote neural stem/progenitor cell (NS/PC) proliferation. NS/PCs-bFGF conduits exhibited similar therapeutic effects to ANT in 8-mm rat facial nerve gaps (Ma et al., 2017). The internal environment of collagen nerve conduits suitable for stem cell growth needs to be further optimized.

#### 4.4.2 Growth factors

Growth factors are involved in the regulation of various cellular processes. Combining collagen-based nerve conduits with growth factors could create a more appropriate microenvironment for nerve regeneration. Recently, multiple concepts have focused on collagen as a nerve conduit with growth factors for nerve regeneration (Table 2). growth could be immobilized on collagen by chemical conjugation (Ma et al., 2018), heparin cross-linking (Ma et al., 2017; Zhang et al., 2021c), and fusion binding domain, such as the laminin-binding domain and collagen-binding domain (Cao et al., 2013). At present, the use of recombinant DNA technology to fuse a binding domain at the N/C-terminus of growth factors is a more popular method (Fujimaki et al., 2020).

**TABLE 2:** Growth factors fill in collagen material to repair PNI.

Growth factors	Mechanism	Strategies	Outcomes	Nerve	Animal models
Basic fibroblast growth factor (bFGF)	• Promotes neurite extension (Fujimoto et al., 1997)	• Linear ordered collagen scaffold (LOCS) filled with collagen binding bFGF (CBD-bFGF) (Ma et al. 2014a, Cui et al. 2014)	• Sustained release (Ma et al. 2014a, Cui et al. 2014)	• Sciatic nerve (Ma et al. 2014a, Fujimaki et al., 2017)	• Rat (Ma et al. 2014a, Fujimaki et al., 2017)
	• Stimulates SCs proliferation (Wang et al., 2003)	• Oriented collagen tubes (Fujimaki et al., 2017)	• Guide axon growth, promote nerve regeneration and functional restoration (Ma et al. 2014a, Cui et al. 2014)	• Facial nerve (Cui et al. 2014, Wang et al. 2020)	• Minipigs (Cui et al. 2014)
		• Collagen conduits filled with CBD- bFGF (Wang et al., 2020)	• Improved nerve repair and recovery of motor function (Fujimaki et al., 2017)		• Rabbit (Wang et al., 2020)
		• Combine with CNTF (Cui et al., 2014)	• Promote functional facial nerve recovery (Wang et al., 2020)		
			• Synergistic effect (Cui et al., 2014)		
Ciliary neurotrophic factor (CNTF)	• Stimulate neurite outgrowth (Hartnick et al., 1996)	• LOCS filled with laminin binding CNTF (LBD-CNTF) (Cao et al., 2011)	• Sustained release (Cao et al. 2011, Cao et al. 2013)	• Sciatic nerve (Cao et al., 2011)	• Rat (Cao et al. 2011, Cao et al. 2013)
	• Increase the number of elongating axon tips (Sahenk et al., 1994)	• LOCS filled with CBD-CNTF (Cui et al. 2014; Lu et al., 2015) or LBD-CNTF (Cao et al., 2013)	• Guide axon growth, promote nerve regeneration and functional restoration (Cao et al., 2011)	• Facial nerve (Cao et al. 2013, Cui et al. 2014, Lu et al. 2015)	• Minipigs (Cui et al. 2014, Lu et al. 2015)
	• Promote neurotransmitter synthesis (Cao et al., 2011)	• Combine with bFGF (Cui et al. 2014), BDNF (Cao et al. 2013)	• Guide axon growth and improve nerve functional recovery (Cao et al., 2013)		
			• Improved axon regeneration, SCs cell migration, remyelination and recovery rate (Lu et al., 2015)		
			• Synergistic effect (Cao et al. 2013, Cui et al. 2014)		
Brain-derived neurotrophic factor (BDNF)	• Promote neuronal growth and differentiation (Yang et al., 2015)	• LOCS filled with CBD-BDNF or LBD-BDNF; Combine with CNTF (Cao et al., 2013)	• Sustained release (Cao et al., 2013)	• Facial nerve (Cao et al., 2013)	• Rat (Cao et al., 2013)
	• Neuronal survival (Hartnick et al., 1996)		• Guided axon growth, promote functional restoration (Cao et al., 2013)		
	• Neuronal plasticity (Castren and Kojima, 2017)		• Synergistic effect (Cao et al., 2013)		
Glial cell line-derived neurotrophic factor (GDNF)	• Promote axonal elongation (Madduri et al., 2009)	• Collagen nerve conduits coated with layers of PLGA (Madduri et al., 2010b)	• Sustained release (Madduri et al. 2010b, Ma et al. 2018, Zhuang et al. 2016)	• Sciatic nerve (Madduri et al., 2010b, Zhuang et al. 2016)	• Rat (Madduri et al. 2010b, Zhuang et al. 2016, Ma et al. 2018)
	• Promote survival of both motor and sensory nerves (Ma et al., 2018)	• Bilayer collagen conduit filled with GDNF-loaded microspheres (Zhuang et al., 2016)	• Improved axonal outgrowth and Schwann cell migration (Madduri et al., 2010b)	• Facial nerve (Ma et al., 2018)	
		• Immobilized on collagen nerve conduits by chemical conjugation (Ma et al., 2018)	• Axonal regeneration and functional recovery similar to autograft (Zhuang et al., 2016, Ma et al. 2018)		
		• Combine with NGF (Madduri et al., 2010b)	• Synergistic effect (Madduri et al., 2010b)		
Nerve growth factor (NGF)	• Support neuron survival and direct neurite outgrowth (Kemp et al., 2011)	• Longitudinally oriented collagen conduit (Yao et al., 2018)	• Improved recovery of regenerated axons and muscle weight (Yao et al., 2018)	• Sciatic nerve (Madduri et al. 2010b, Yao et al. 2018)	• Rat (Madduri et al., 2010b)
	• Induced extensive axonal branching (Madduri et al., 2009)	• Combine with GDNF (Madduri et al., 2010b)	• Synergistic effect (Madduri et al., 2010b)		• Dog (Yao et al., 2018)
	• Regulate the receptivity of axons to myelination (Chan et al., 2004)				
Vascular Endothelial Growth Factor (VEGF)	• Vasculogenesis and angiogenesis (Lu et al., 2019)	• Collagen tube filled with CBD-VEGF-immobilized collagen Fibers (Ma et al., 2014b)	• Sustained release (Ma et al., 2014b)	• Sciatic nerve (Ma et al., 2014b)	• Rat (Ma et al., 2014b)
	• Induced extensive neurite growth and branching (Guaiquil et al., 2014)		• Guided axon growth, morphological and functional improvements similar to autograft (Ma et al., 2014b)		

##### 4.4.2.1 Basic fibroblast growth factor

The bFGF is a member of the fibroblast growth factor family and is abundantly expressed in neural tissue. It played a crucial role in the mitogenesis and proliferation of SCs (Davis and Stroobant, 1990) and DRG neurons *in vitro* (Li et al., 2002) and stimulated persistent angiogenesis *in vivo* (Chu et al., 2011). Moreover, bFGF is involved in the early peripheral nerve regeneration by activating autophagy to accelerate myelin debris clearance (Li et al., 2020), and further *in vivo* study confirmed that the regulation of bFGF in autophagy was achieved by activating the PAK1 pathway in SCs (Hu et al., 2022). Therefore, it is considered as a growth factor that is beneficial for nerve regeneration. *In vivo* studies have confirmed the effect of collagen conduits combined with bFGF on axonal regeneration and functional recovery (Fujimaki et al., 2017). However, bFGF cannot play a long-term effect after transplantation due to its poor immobilization on collagen nerve conduits, so various strategies have been proposed to ensure its sustained release *in vivo*. Based on its high affinity for heparin (Sakiyama-Elbert and Hubbell, 2000; Yang et al., 2010), bFGF was immobilized on nerve conduits cross-linked with heparin, which facilitated the local sustained release of bFGF *in vivo* (Ma et al., 2017). A novel approach to improving the affinity of bFGF for collagen was also previously reported. The N-terminal of native bFGF was fused with a CBD, TKKTLRT, which could specifically bind to collagen (Li et al., 2011; Ma et al., 2014a). The sustained release of the bFGF further improved nerve regeneration *in vivo* (Cui et al., 2014; Wang et al., 2020).

##### 4.4.2.2 Neurotrophins

Neurotrophins are a protein family associated with the survival of sensory and sympathetic neurons. They participate in regulating various aspects of neuronal development and function (Reichardt, 2006). Damage to peripheral nerves induces active cellular mechanisms that lead to the synthesis of neurotrophins in neurons and SCs to promote nerve regeneration (Richner et al., 2014). The two main receptors were tropomyosin receptor kinase (TrkA, Trk-B, and Trk-C) and P75NTR. Neurotrophins could activate the downstream targets of various signaling cascades by binding with their corresponding receptors to exert their multiple effects on neurorestoration. The signaling pathways involved in neurotrophins after nerve injury include PI3K/Akt, MAPK/ERK, JNK/c-Jun, and Rho A/ROCK (Li et al., 2020). Various neurotrophins were loaded on collagen scaffolds to investigate their promoting effects on peripheral nerve regeneration, such as brain-derived neurotrophic factor (BDNF) (Cao et al., 2013), ciliary neurotrophic factor (CNTF) (Cao et al., 2011; Lu et al., 2015), nerve growth factor (NGF) (Yao et al., 2018; Long et al., 2021), GDNF (Zhuang et al., 2016). To ensure the sustained release of neurotrophins *in vivo*, LBD (Cao et al., 2011), CBD (Cui et al., 2014), and chemical conjugation (Ma et al., 2018) were proposed for their immobilization on collagen. The combination of neurotrophins may have a synergistic effect on nerve regeneration. For instance, GDNF and NGF were functionally complementary in promoting axonal elongation and axonal branching (Madduri et al., 2009). Their combination exerted a synergistic effect on axonal growth (Madduri et al., 2010a; Madduri et al., 2010b). The combination of CNTF and BDNF within LOCS showed enhanced facial nerve regeneration and functional recovery (Cao et al., 2013). Thus, combining multiple neurotrophins and collagen nerve conduits may be a better treatment to repair PNI in the future.

## 5 Discussion

Nerve conduits prepared from several biomaterials offer a promising approach to promoting peripheral nerve regeneration, such as the natural polymers collagen, chitosan, and silk and the synthetic polymers poly-ε-caprolactone (PCL), poly-lactic-co-glycolic acid (PLGA), and poly-glycolic acid (PGA) (Pinho et al., 2016). Compared with synthetic biomaterials, natural polymeric proteins exhibit more excellent biocompatibility and biodegradability, and evoke a minimal inflammatory reaction after transplantation (Jiang et al., 2020a). Thus, natural polymers have attracted great attention in tissue engineering. The peripheral nerve is surrounded by ECM *in vivo*, and its repair is a complicated process that requires cell-cell and cell-ECM interactions. Thus, simulating neural tissue in the natural microenvironment is an important factor in designing nerve conduits (Sarker et al., 2018). As the major component in the ECM of peripheral nerve, the fibrous structure of collagen can simulate native nerve tissue, creating a suitable microenvironment for nerve regeneration (Hosseinkhani et al., 2013). Compared with other natural polymers, collagen exhibited higher affinity for nerve cells (Cheng et al., 2003). Its semipermeable membrane structure allows the exchange of nutrients and metabolites while preventing the outward migration of cells within the conduits. In addition, collagen is the major component of FDA-approved artificial nerve conduits for the surgical reconstruction of the peripheral nerve due to its low antigenicity and immunogenicity (Kornfeld et al., 2019). Commercially available collagen nerve guides showed potential therapeutic effects for bridging larger nerve gaps (Bozkurt et al., 2017) and functional recovery in the clinic (Wangensteen and Kalliainen, 2010). Therefore, collagen is an extensively used natural polymer in preclinical *in vivo* studies, second only to chitosan (Gregory and Phillips, 2021). Collagen is considered a promising material for the preparation of nerve conduits as an alternative to autografts for PNI.

Over recent years, collagen nerve conduits have evolved from single hollow conduits to functional composite nerve conduits. Collagen nerve conduits loaded with bioactive components have shown improved biological performance due to mimicking biological processes following nerve injury *in vivo* (Carriel et al., 2013; Zhuang et al., 2016; Ma et al., 2017). However, some concerns remain to be considered. The emergence of stem cells overcame the poor proliferation and differentiation capacity of SCs, and collagen nerve conduits loaded with differentiated stem cells could induce more sciatic motoneurons regenerating axons *in vivo* (Ladak et al., 2011). However, nonscalable protocols control the transdifferentiation of stem cells. It is challenging to maintain the differentiated state of the stem cells *in vitro* under dynamic *in vivo* conditions (Uz et al., 2018). More efficient and scalable transdifferentiation procedures are required to precisely control the final fate of implanted stem cells loaded in collagen nerve conduits. In addition, although the sustained release of growth factors *in vivo* has been greatly improved by CBD, LBD, or chemical conjugation, more precise control methods still need to be investigated to control the release of growth factors in different stages of peripheral nerve repair. Furthermore, the combination of growth factors and supportive cells in collagen nerve conduits showed synergistic effects on PNI. For instance, anchored bFGF on collagen exhibited sustained mitogenic and anti-apoptotic effects on NS/PCs, the combination of bFGF and NS/PCs showed synergistic therapeutic effect in the restoration of facial nerve defects, similar to ANT (Ma et al., 2017). NGF is involved in stem cell growth. Incorporating HuMSCs and NGF further enhanced the repair effect of collagen scaffolds on recurrent laryngeal nerve injury (Pan et al., 2017). Thus, the combination of various bioactive components is a promising therapeutic strategy.

In conclusion, we summarize the characteristics of collagen as biomaterial and the roles of extensively studied collagen types in peripheral nerve regeneration. The optimization of collagen nerve conduits in terms of physical properties, structure and combination of bioactive components was further investigated. With the successive proposals of various therapeutic strategies, the therapeutic effects of collagen nerve conduit have been greatly improved, even similar to ANT (Ma et al., 2017; Burks et al., 2021; Long et al., 2021). However, it should be mentioned that the major clinical challenge is the repair of large nerve gaps. There are only a few studies on applying collagen nerve conduits in large animal models of PNI (Cui et al., 2014; Cui et al., 2018). Compared with rodent models, large animal models can be used to create longer gaps for preclinical evaluation, which is more closely applicable to the challenge of artificial nerve conduits in PNI. Therefore, the application of large animal models to evaluate the effects of functional collagen nerve conduits on peripheral nerve regeneration may accelerate the transition to the clinic in the future.
